# Correction: Nutritional status of Indian adolescents (15-19 years) from National Family Health Surveys 3 and 4: Revised estimates using WHO 2007 Growth reference

**DOI:** 10.1371/journal.pone.0239923

**Published:** 2020-09-24

**Authors:** Madhavi Bhargava, Anurag Bhargava, Sudeep D. Ghate, R. Shyama Prasad Rao

There are errors in the Funding statement. The correct Funding statement is as follows: MB received a grant from The United Nations Children's Fund (UNICEF) for a capacity building workshop on Nutrition Assessment Techniques. The URL of the website is: https://unicef.in/Karnataka. UNICEF is acknowledged for their support towards the Center for Nutrition Studies, Yenepoya (Deemed to be University). No additional external funding was received for this study.
