# Supplementary material for: Nutritional status of Indian adolescents (15-19 years) from National Family Health Surveys 3 and 4: Revised estimates using WHO 2007 Growth reference
Source: PLoS One. 2020 Jun 22;15(6):e0234570. doi: 10.1371/journal.pone.0234570 (PMC7307767; doi:10.1371/journal.pone.0234570)
Supplement: Attachment — Submitted filename: 5. Response to reviewers.docx [file pone.0234570.s002.docx]

Reviewer #1: The manuscript is very well written and fulfils the gap in anthropometric data analysis for adolescents in NFHS - 3 and NFHS - 4. All tables and graphs have been presented comprehensively and does complete justice for analysis of demographic data.

**RESPONSE:**

We thank you for your encouraging comments.

Reviewer #2: The manuscript is well written and logically presented. It throws light on the nutritional status of the adolescent age group that is often sidelined. The manuscript also has shown the right method of classification of nutritional status and adolescent age group with use of appropriate standards for assessment of nutritional status.

**RESPONSE:**

Thank you for your positive comments.

Reviewer #2: Suggestion to the authors: will there be a difference in classification of nutritional status of the adolescent if IAP classification is used instead of WHO?

**RESPONSE:**

Authors agree that older children and adolescents have growth patterns which may be different in different populations of the world. This is because nutritional, environmental, genetic factors and timing of puberty seem to play a major role not only in the attainment of final height but also in the characteristics of the growth curve.

It might be a good idea to analyze this data using the IAP charts also to see the difference in estimates, but it is beyond the scope and objective of this manuscript.

While the IAP charts are very useful in individual surveys, presently there is no software/algorithm available based on the IAP charts that can analyze big data like that in NFHS. But the IAP estimates would definitely be closer to our WHO estimates than those using the adult cut-offs (presently reported by the NFHS). The advantage of using WHO growth reference is that it avails comparability between countries.

Authors thank you for this valuable suggestion and we may think of doing this exercise as a new project.
